# A Cellulose/Laponite Interpenetrated Polymer Network (IPN) Hydrogel: Controllable Double-Network Structure with High Modulus

**DOI:** 10.3390/polym10060634

**Published:** 2018-06-08

**Authors:** Fan Xie, Cécile Boyer, Victor Gaborit, Thierry Rouillon, Jérôme Guicheux, Jean-François Tassin, Valérie Geoffroy, Gildas Réthoré, Pierre Weiss

**Affiliations:** 1 Regenerative Medicine and Skeleton (RMeS), INSERM UMR_S1229, Université de Nantes, Centre Hospitalier Universitaire de Nantes, ONIRIS, F-44042 Nantes, France; fan_xie@huntsman.com (F.X.); cecile.boyer@univ-nantes.fr (C.B.); victor.gaborit@etud.univ-angers.fr (V.G.); thierry.rouillon@univ-nantes.fr (T.R.); Jerome.Guicheux@univ-nantes.fr (J.G.); valerie.geoffroy@univ-nantes.fr (V.G.); gildas.rethore@univ-nantes.fr (G.R.); 2 CNRS UMR6283, Institut des Molécules et Matériaux du Mans (IMMM), Le Mans Université, F-72000 Le Mans, France; Jean-Francois.Tassin@univ-lemans.fr; 3 School of Dentistry, Université de Nantes, F-44042 Nantes, France; 4 Nantes University Hospital, CHU Nantes, PHU4 OTONN, F-44042 Nantes, France

**Keywords:** hydrogel, clay, biomaterial, tissue engineering, cytotoxicity, interpenetrating networks, composite hydrogels

## Abstract

Laponite XLS™, which is a synthetic clay of nanometric dimensions containing a peptizing agent, has been associated with silanized hydroxypropylmethylcellulose (Si-HPMC) to form, after crosslinking, a novel composite hydrogel. Different protocols of sample preparation were used, leading to different morphologies. A key result was that the storage modulus of Si-HPMC/XLS composite hydrogel could be increased ten times when compared to that of pure Si-HPMC hydrogel using 2 wt % of Laponite. The viscoelastic properties of the composite formulations indicated that chemical and physical network structures co-existed in the Si-HPMC/XLS composite hydrogel. Images that were obtained from confocal laser scanning microscopy using labelled Laponite XLS in the composite hydrogels show two co-continuous areas: red light area and dark area. The tracking of fluorescent microspheres motions in the composite formulations revealed that the red-light area was a dense structure, whereas the dark area was rather loose without aggregated Laponite. This novel special double-network structure facilitates the composite hydrogel to be an adapted biomaterial for specific tissue engineering. Unfortunately, cytotoxicity’s assays suggested that XLS Laponites are cytotoxic at low concentration. This study validates that the hybrid interpenetrated network IPN hydrogel has a high modulus that has adapted for tissue engineering, but the cell’s internalization of Laponites has to be controlled.

## 1. Introduction

Hydrogels have been studied for a variety of applications these years [1,2,3,4,5]. The hydrophilic three-dimensional network structure of hydrogels is able to retain large amounts of water, which gives ability to change shape and volume in response to external stimuli. The good swelling properties also give hydrogels wide applications, such as biosensors, drug delivery, and tissue engineering. Hydroxypropylmethylcellulose (HPMC) is a commercial semisynthetic cellulose derivative that can be dissolved in water to form hydrogels. Weiss et al. [6,7] firstly developed an injectable suspension for tissue engineering by simply mixing biphasic calcium phosphate granules into HPMC solution. The mixture exhibited good biocompatibility and proper rheological properties for convenient injection, but had a tendency to flow after implantation in vivo. Later, the composite was improved by grafting silane on HPMC, leading to a self-cross-linkable hydrogel (silanized hydroxypropylmethylcellulose, Si-HPMC) without using any toxic chemical catalyst [8,9]. The hydrogel (Si-HPMC) has excellent biocompatibility and bioactivity [10,11,12,13], which makes it a promising material for tissue engineering. The storage modulus of the gel can be improved by increasing the cellulose concentration or the crosslinking density, but the latter is limited by synthesis difficulties. 

Laponite is a synthetic layered silicate that can be well dispersed in water. The individual Laponite particle is in the form of disc-shaped crystals of about 25 nm in diameter and 1 nm in thickness [14,15]. Recently, Haraguchi et al. [16] synthesized a nanocomposite hydrogel using Laponite as a cross-linker instead of conventional chemical cross-linkers to form a network structure. The obtained hydrogel was highly stable, structurally homogeneous, and exhibited good mechanical properties. Further investigations showed that the tensile moduli and strengths were almost proportional to the clay content [17,18,19]. In order to increase the clay content in the hydrogel, Liu et al. [20] chose modified Laponite and synthesized a high clay content nanocomposite hydrogel to improve mechanical properties. However, the in-situ radical polymerization precludes such hydrogel from being implanted in vivo. If the hydrogels are prepared in vitro, the injectability of hydrogels will be lost. Moreover, most of the polymer concentrations that are reported in hydrogels was about 10 wt %. Dealing with tissue engineering applications, the concentration of polymer in hydrogel, which is often linked to the network density, is responsible to lower mass transfer properties with less diffusion of nutrients, oxygen, and glucose [21]. In most cases, the lower the concentration of polymer in hydrogel, the better the cytocompatibility of hydrogel. 

In this study, a novel Si-HPMC/Laponite composite hydrogel has been developed. Laponite XLS, abbreviated as XLS in the following, a modified Laponite, was used to reinforce the Si-HPMC hydrogel, according to different protocols of sample preparation. The concentration of Si-HPMC was fixed at a relatively low value of 2 wt % and the XLS content ranged from 0.5 to 2 wt % for the mechanical properties test and up to 5 wt % for cytotoxicity examinations. The gelation processes and the viscoelastic properties of composite hydrogels with different XLS contents were studied by rheological methods. Confocal laser scanning microscopy (CLSM) and fluorescent microspheres were used to investigate the morphology of composite hydrogels and to track the distribution of the components. A special double-network structure was observed in this composite hydrogel. With this special double-network, the mechanical properties of composite hydrogel were significantly improved. To end this study, cytotoxicity’s assays have been performed and suggested that XLS Laponites are cytotoxic, even at low concentration.

## 2. Materials and Methods 

### 2.1. Materials

Hydroxypropylmethylcellulose (HPMC), which was used in this study, was Methocel E4M, natural grade product with a weight-average molecular weight of about 290,000 g/mol, as determined by light scattering [9]. It was purchased from Colorcon Limited (Dartford Kent, England). 3-Glycidoxypropyltrimethoxysilane (GPTMS), which is the group to be grafted onto HPMC, and 4-(2-hydroxyethyl) piperazine-1-ethanesulfonic acid (HEPES), which is used for preparing buffer solution, were supplied by Aldrich (Darmstadt, Germany). The inorganic clay, synthetic hectorite “Laponite XLS”, was provided by Rockwood Additives (Cheshire, UK). The chemical composition of XLS is: SiO_2_ 54.5%, MgO 26.0%, Li_2_O 0.8%, Na_2_O 5.6%, P_2_O_5_ 4.1%, and the density is about 2500 kg/m^3^. 

### 2.2. Preparation of Hydrogels

HPMC was first silanized by grafting GPTMS according to the method described previously [9]. Inductively coupled plasma atomic emission spectroscopy (ICP-AES) was used to determine the amount of grafted silane, and the percentage (*w*/*w*) was calculated as 0.59% [22]. 

As Si-HPMC is only soluble in basic solutions (minimum [NaOH] = 0.1 M), 6 g of Si-HPMC powder were dissolved in 200 ml sodium hydroxide solution ([NaOH] = 0.2 M) at 25 °C with stirring for 48 h. Then, the basic Si-HPMC solution was dialyzed in dialysis bags (Spectra-por 6000–8000) against 3.8 L of NaOH solution (0.09 M) for 16 h and 4 L of NaOH solution (0.09 M) for 2 h. The final pH value of the basic Si-HPMC solution was about 12.7. 

An acidic buffer solution used to neutralize the basic Si-HPMC solution was prepared. Briefly, 6.2 g HEPES were dissolved in an acidic solution containing 1.8 g NaCl and 30 mL HCl (0.2 M). The volume of acidic solution was adjusted to 50 mL with deionized water and was kept stirring for about 15 min. The pH value of the final acidic buffer solution was about 3.6.

The composite hydrogels were prepared according to two different protocols: 

The protocol A (XLS suspension in acidic buffer) is as follows: for each formulation (3), different amounts of XLS powders (0.15, 0.3, and 0.6 g) were first dispersed in 10 g acidic buffer solution and the XLS suspension was adjusted to 20 g with deionized water, followed by continuous stirring for 2 h. 4 mL of basic Si-HPMC solution were blended with 2 mL of the previous acidic XLS suspension in order to obtain a composite hydrogel formulation with pH of 7.4.

The protocol B (XLS suspension in neutral aqueous water) is as follows: for each formulation (3), different amount of XLS powders were first dispersed in deionized water and the mixture was stirred for 2 h to get transparent dispersions with XLS concentrations of 3, 6, and 12 wt %, respectively. Secondly, 4 mL basic Si-HPMC solution were blended with 1 mL acidic buffer solution to obtain a neutral hydrogel solution. Then, this neutral hydrogel solution was blended with 1 mL XLS dispersion and the composite hydrogel was obtained. 

Different concentrations of XLS lead to composite hydrogels with 0.5, 1, and 2 wt % of XLS in the both protocols. 

Pure unfilled Si-HPMC hydrogel was obtained when neutral hydrogel solution (basic Si-HPMC solution blended with acidic buffer before cross-linking) was added to 1 mL deionized water. If HPMC was used instead of Si-HPMC, HPMC/XLS composite hydrogels with different content of XLS could also be obtained following the same preparation protocols.

### 2.3. Rheological Measurements

As the samples undergo a rapid gelation in our experiments, the whole time used for blending different aqueous solutions was limited to 3 min. Then, the blended solutions were placed on a rotational rheometer (RheoStress 300, Thermo Haake Co., Karlsruhe, Germany) and the rheological properties of samples were monitored in different modes under oscillatory shear with a selected titanium cone-plate geometry (60 mm diameter, 1° cone angle). A ring with oil was used to cover the free surface of the sample in order to prevent the evaporation of solvent during measurements. The stress amplitude was fixed at 0.5 Pa, which lies in the linear viscoelastic region, and the angular frequency was 1 rad/s. The temperature was set at 37 °C during the test. 

### 2.4. Morphology Characterization

A confocal laser scanning microscope (TCS-SP2, Leica Microsystems, Heidelberg, Germany) was used to investigate the dispersion of Laponite in the composite hydrogels, and Auramine O was chosen as fluorescent probe to label XLS. Briefly, XLS powders were dispersed in Auramine O solution with a concentration of 30 ppm and the dispersion was stirred for about 2 h in order to get light yellow transparent labelled-XLS dispersion. Then, the labelled-XLS dispersion was dialyzed against deionized water for about 12 h to remove free Auramine O molecules. Finally, the dialyzed labelled-XLS dispersion was used to blend with neutral hydrogel solution to form composite hydrogel, as mentioned before. A water immersion objective lens was used to observe the sample. The incident light was emitted by a laser beam at 488 nm and the fluorescence light was recorded at 510 nm. 

In order to observe the structure inside the composite hydrogels, fluorescent microspheres (Fluoresbrite^®^ Polychromatic Red Microspheres 0.5 μm, Polysciences, Inc., Warrington, PA, USA) were used and the final concentration in the composite formulations were adjusted to about 5 ppm. All of the samples with fluorescent microspheres were observed under CLSM 2 h after preparation. Multi-incident light was emitted by the laser beam at 488 nm and 543 nm, and the fluorescence light was recorded at 510 nm and 560 nm. In general, images contain around 50 particles. An image size of 250 mm was considered. Trajectories of the particles were monitored for different time intervals between images. The smallest was 0.3 s. In all cases, 200 8-bit tiff images of 512 × 512 pixels were recorded and combined to make a film of the particle displacements. Motions were recorded up to 100 s. The movies were analyzed with a particle tracking routine that was developed by J. C. Cocker and E. R. Weeks. The diffusion coefficient was determined from 5 films taken from different positions in the gel.

This setup can distinguish fluorescent microsphere from dyed Laponite by different colours. 

### 2.5. Cell Viability

Cell culture: prior any biocompatibility experiments, Si-HPMC basic solution, and XLS solutions were autoclaved at 120 °C during 20 min with an Alphaklave 23.

Human chondrosarcoma cell line (SW1353, ATCC, Molsheim, France) was cultured in a 5% CO_2_ incubator at 37 °C in Dulbecco’s modified Eagle’s medium (DMEM, Invitrogen Corp., Carlsbad, CA, US). Culture media was supplemented with 10% fetal bovine serum, 1% penicillin/streptomycin and 1% L-glutamine, and changed every 2–3 days.

Cell viability in two-dimensions (2D): two different experiments have been performed, (1) evaluation of SW1353 cell viability cultured in direct contact with increasing concentrations of XLS and (2) evaluation of SW1353 cell viability cultured in contact with Si-HPMC hydrogel containing increasing concentrations of XLS. SW1353 cells were allowed to attach to 24-well plates at a final density of 10,000 cells per cm^2^. After 24 h, the culture medium was removed and either XLS or Si-HPMC containing XLS were added onto the cell layer. For the culture in direct contact, 500 µL of culture medium containing 0.001, 0.01, 0.1, or 1% XLS (% *w*/*v*) were added to each well and refreshed every two days. For the culture in contact with hydrogel 500 μL/well of precursor solution of pure Si-HPMC or of each nanocomposite were added. The hydrogel samples were incubated at 37 °C for 1 h before adding 1 mL of culture medium. As a positive control, cells were cultured in the absence of Si-HPMC hydrogel. As a negative control, cells were cultured in the presence of actinomycin-D (5 μg mL^−1^), a transcription inhibitor. After 1, 2, 4, and 6 days of culture the hydrogels and culture medium were removed by aspiration. Trypan blue exclusion dye assays were performed to count the cells. In order to evaluate their mitochondrial activity, the methyl tetrazolium salt (MTS) (Promega, Madison, WI, USA) test was performed by adding the MTS solution to each well for 1 h. The optical density of the formazan dye was measured in a spectrophotometer at 490 nm. Each condition was tested in quadruplicate.

Cell viability in three-dimensions (3D): 3D culture cell viability was assessed by using Live&Dead Assay Kits (Invitrogen, Carlsbad, CA, US) along with confocal image analysis. SW1353 cells were dispersed in the hydrogel precursor solutions within 5 min, following their preparation (thus before gelification) at a final concentration of 1,000,000 cells per ml of hydrogel. Five hundred microliters of each mixture were molded into 24-wells plates and incubated at 37 °C for 1 h to allow for the gelification of the hydrogels. Afterwards, 1 ml of DMEM medium was added per well and the samples were incubated for 1, 2, and 6 days before the Live&Dead assays. Actinomycin-D treatment (5 μg mL^−1^) was used as an internal control for cell death. In each well, the culture medium was replaced by 200 μl of a solution containing 2.5 mL of DMEM medium that was supplemented with 0.25 μL of calcein-AM and 5 μL of ethidium homodimer-1. After 45 min, the dye mixture was removed and the hydrogels were intensively rinsed with phosphate buffered saline solution before being observed with a confocal laser scanning microscope (Nikon D-eclipse C1, Champigny sur Marne CEDEX, France)), equipped with an argon/krypton laser. Each condition was tested in quadruplicate and for each sample two random positions (x, y, z) were chosen within the hydrogel and a series of images were recorded starting from these positions along the z-axis. The 210 images that were obtained per sample were analyzed with a quantimeter Q550 (Leica Microsystems, Wetzlar, Germany).

### 2.6. Transmission Electron Microscopy (TEM):

For TEM experiments with cells and Laponites, adherent chondrosarcoma cells (SW1353) were first grown until confluence in multiwell culture plates before being incubated during 24 h or 48 h with DMEM media containing 1% (*w*/*v*) of Laponites. After incubation, cells were rinsed with PBS and were detached with a trypsin solution. After gentle centrifugation, the cells were fixed several days at 4 °C with a 1.6% of glutaraldehyde in 0.1 M cacodylate buffer solution (pH 7.4), then rinsed several times with a 0.1 M cacodylate buffer solution (pH 7.4), and post-fixated during 45 min at room temperature with a 1% osmium tetroxide in 0.1 M cacodylate buffer solution. After post-fixation, cells were rinsed several times with water and were dehydrated in gradually concentrated baths of ethanol and then of propylene oxide before being impregnated and embedded in EPON resin. Blocks containing cells were cut in thin layers (<100 nm) by ultramicrotomy (Ultracut E, Reichert-Jung) with a diamond knife and deposited on copper grids that were previously covered with a thin collodion film. Grids were then contrasted with lead citrate and uranyl acetate solutions before being observed. The TEM observations were performed in the conventional bright field mode with a transmission electron microscope (JEM 1010, JEOL, Akishima, Japan), operating at 80kV and equipped with a four Megapixels retractable digital CCD camera (ORIUS^TM^ SC200W, GATAN, Sarasota, CA, US).

### 2.7. Statistical Analysis

The results are expressed as mean ± SEM. Statistical analysis was carried out using two-way ANOVA. Where significant overall differences were detected by ANOVA, Fisher’s test was used for further difference comparison as sample sizes are small. *p* values of less than 0.05 were considered to be significant. The statistical analysis program used was Statview (SAS Institute, Cary, NC, US).

## 3. Results

### 3.1. Gelation Process

HPMC or Si-HPMC is white powder in appearance and it only dissolves in basic solution, as mentioned before. When the basic Si-HPMC solution is blended with acid buffer solution, the mixture is neutralized and the pH value decreases to about 7.4, which causes sodium silanolate transformation into silanol and leads to a condensation reaction [9]. 

**Figure 1:**
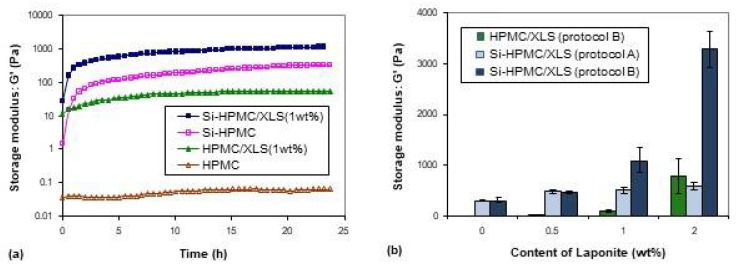
(**a**) Evolutions of storage modulus of different systems with time. Composite hydrogels were prepared using B. (**b**) Effects of XLS Laponites content on the final storage moduli of composite hydrogels prepared using different protocols after one day. Each data point represents mean ± SEM for n ≥ 3 samples.

Typical evolution of the storage modulus during one day are shown in Figure 1a. The pure HPMC solution remained at low modulus in the whole day, indicating that the macromolecules are still in solution. However, the storage modulus of the other three formulations increased sharply, especially when Si-HPMC is used, indicating that some network structures are formed. It can be noticed that the increase of storage modulus were different between HPMC and Si-HPMC formulations (samples were prepared using protocol B).

Figure 1b shows the final storage moduli of different hydrogel formulations after one day. The storage moduli of hydrogel composites increased with increasing contents of XLS mostly with protocol B, showing that the addition of Laponite indeed improved the storage moduli of the hydrogel formulations using the protocol B. It should also be noticed that the protocol B controlled the different final storage moduli, regardless of the content of Laponite. At low content of Laponite (≤0.5 wt %), the final moduli of Si-HPMC/XLS formulations that were prepared with the two protocols were not significantly different. However, when the content of Laponite was higher (1, 2 wt %, and above), protocol B was more efficient than protocol A to increase the moduli of the gels. The value of Si-HPMC/XLS formulation modulus with 2 wt % of XLS was even ten times higher than that of pure Si-HPMC formulation, showing the reinforcement that was brought by Laponite. 

### 3.2. Viscoelastic Properties

**Figure 2:**
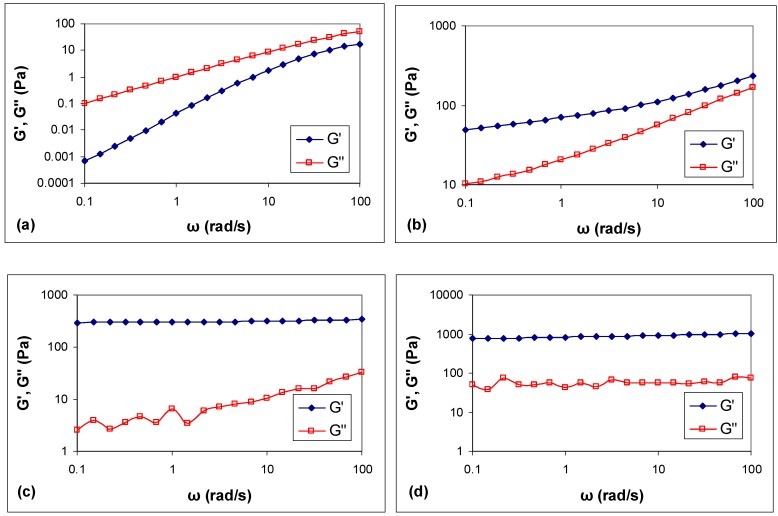
Storage modulus, G’, and loss modulus, G”, of different samples as function of frequency after one day at 37 °C. (**a**) Hydroxypropylmethylcellulose (HPMC); (**b**) HPMC/XLS; (**c**) Si-HPMC; (**d**) Si-HPMC/XLS. The content of XLS in composite hydrogels, which were prepared using B, were 1 wt %.

On the basis of the nature of their crosslinks, hydrogels can be classified as chemical hydrogels when the crosslinks are covalent bonds, or as physical hydrogels when the links are secondary weak bonds, such as Van der Waals, electrostatic interactions, hydrogen bonds, or molecular entanglements. It is possible to distinguish them from mechanical spectra. Figure 2 shows the frequency sweep results of different hydrogels prepared using B and tested after 24 h gelation at 37 °C. 

For HPMC solution (Figure 2a), G” is much higher than G’ throughout the test frequency range and G” ≈ ω^1^ meaning that the HPMC is in a viscous solution. For HPMC/XLS formulation (Figure 2b), the profile of G′ and G″ moduli showed a mild frequency dependence, especially at low frequencies and the ratio G’/G” was lower than 10, indicating a typical weak gel. For Si-HPMC formulation (Figure 2c), G’ is much higher than G” throughout the test frequency range and it is independent on frequency, revealing a rather strong gel [23]. For Si-HPMC/XLS formulation (Figure 2d), G′ and G″ curves are independent of the frequency and parallel to each other, showing almost horizontal straight lines. Moreover, G′ values are 1 to 2 orders of magnitude greater than G″ values, which is also typical of a strong gel. Note however that the damping at low frequency is higher in the presence of XLS particles.

**Figure 3:**
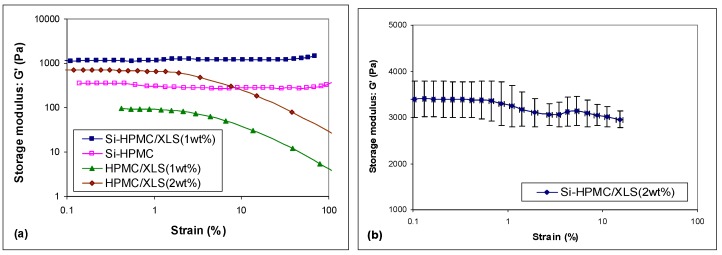
Storage modulus of (**a**) HPMC/XLS, pure Si-HPMC, Si-HPMC/XLS (1 wt %), and (**b**) Si-HPMC/XLS (2 wt %) composite hydrogel as function of strain after one day at 37 °C. The composite hydrogels were prepared using B and results represent mean ± SEM for n ≥ 3 samples.

The critical deformation γ_0_ characterizing the limit of the linear viscoelastic region also allows for us to discriminate the different formulations. Figure 3 shows the strain sweep curves of different hydrogels that were prepared using B and tested after 24 h gelation at 37 °C. Both pure Si-HPMC and Si-HPMC/XLS composite hydrogels show steady value in the entire test range, meaning that the linear viscoelastic regime extends over 100% strain. On the contrary, the linear regime is limited to deformations below 1% for HPMC/XLS hydrogels, which is typical of weak physical gel structures [24]. Figure 3b shows the amplitude sweep curve of Si-HPMC/XLS composite hydrogel with 2 wt % XLS prepared using B. A slight drop of modulus is observed at about 3% strains, which lies in the same area as the critical deformation of the HPMC/XLS composite hydrogel.

### 3.3. Morphology

In order to observe the morphology of these complex systems, confocal laser scanning microscopy (CLSM) was used to investigate the dispersion of Laponite in the composite hydrogels.

**Figure 4:**
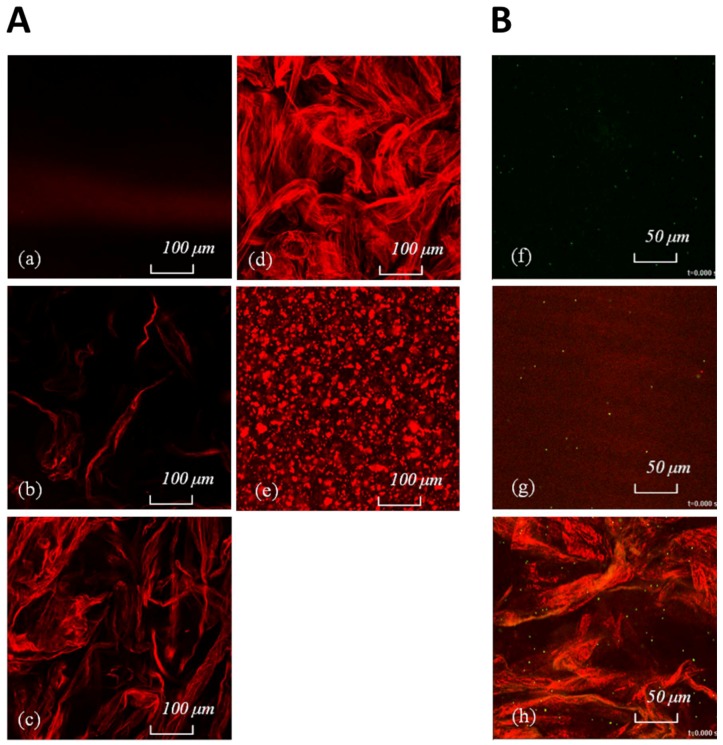
**A**. Confocal laser scanning microscopy (CLSM) images of (**a**) XLS suspension whose concentration is 2 wt % and Si-HPMC/XLS composite hydrogels prepared using protocol B with the contents of XLS being (**b**) 0.5 wt %, (**c**) 1 wt %, and (**d**) 2 wt %, respectively. Laponite particles are stained by auramine o and appear in red, provide the local concentration is high enough. Images are thus revealing the aggregation of Laponite. CLSM image of Si-HPMC/XLS (1 wt %) composite hydrogel prepared using protocol A is shown in (**e**). **B**: CLSM images of different systems with fluorescent microspheres (green dots) taken from movies (supplementary data) are shown in (**f**–**h**). XLS appears red and fluorescent microspheres appear green under CLSM. (**f**) Physiological saline solution; (**g**) XLS suspension in physiological saline; and (**h**) HPMC/XLS composite hydrogel. The images of HPMC, Si-HPMC are similar to that of (**f**); the image of XLS suspension without ionic strength is similar to that of (**g**); and, the image of Si-HPMC/XLS composite hydrogel is similar to that of (**h**) (data no shown). All of the images were taken after samples were prepared for 2 h.

Figure 4b–d shows images of Si-HPMC/XLS composite hydrogels with increasing XLS contents. The images of HPMC/XLS composite formulations are similar to that of the silanized ones (data not shown). As Laponite is well dispersed in the aqueous solution and the dimension of a single particle is less than 50 nm, nothing can be observed under CLSM, as shown in Figure 4a, even though particles were labelled with fluorescent dye. When the Laponite was added to the hydrogel, XLS individual particles began to aggregate and turned to be visible. It can be seen from Figure 4b–d that the aggregated masses are in red as expected, but they are separated when the concentration of XLS is as low as 0.5 wt % (Figure 4b). When the content of XLS in hydrogels increased, the aggregated masses began to form a continuous physical network, as shown in Figure 4c,d. In contrary, using the protocol A no continuous physical network is obtained, but rather compact separated aggregates of Laponite (Figure 4e).

### 3.4. Structure Inside

As particles can make Brownian motion in fluids and this random movement can be recorded and described by mathematical models, fluorescent microspheres have been added in the composite hydrogels and the movements of these fluorescent microspheres have been recorded by CLSM. The root mean square displacement of microspheres moving in any direction after time t can be expressed, as following [25]:

(1) $$\bar{x}=\sqrt{2Dt}$$

in which *D* is diffusion coefficient. *D* is related to viscosity *η* of the surrounding matrix through the radius *r* of the moving particle (in our case fluorescent microspheres) [26]:

(2) $$D=\frac{RT}{6\pi N_{a}\eta r}$$

in which *R* is gas coefficient, *T* is absolute temperature, and *N_a_* is Avogadro’s constant. Using Equations (1) and (2), the local viscosity of matrix surrounding the moving particles can be calculated from displacements that were recorded by CLSM. 

**Figure 5:**
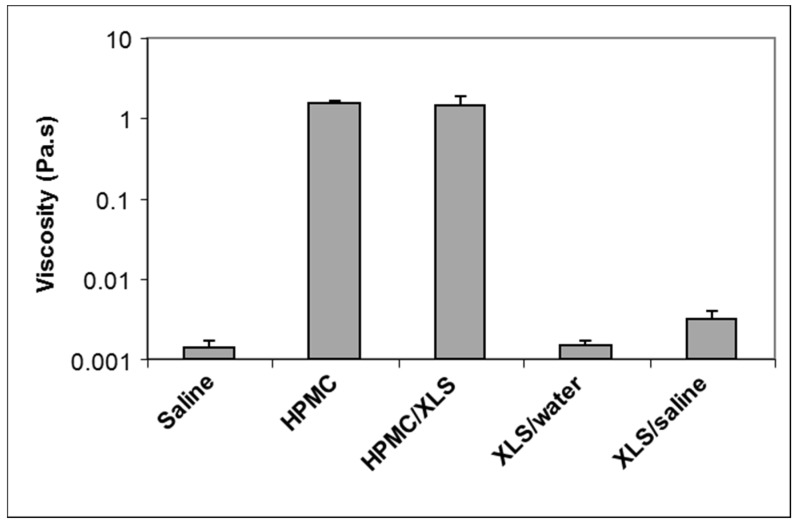
The viscosities of matrices surrounding fluorescent microspheres in different systems. For HPMC/XLS system, matrix means the loose part in dark area. Each data point represents mean ± SEM for n ≥ 3 samples.

Figure 4f–h shows some CLSM images of fluorescent microspheres in different formulations extract from video (supplementary data Video 1–8). Brownian motions of fluorescent microspheres in different formulations can be observed in video recorded by CLSM and the local viscosity measured in different formulations, 2 h after mixing, is shown in Figure 5. 

The Brownian motions of fluorescent microspheres were visible in physiological saline and in XLS suspension in deionized water, and the viscosities that were calculated from microspheres movements in these two formulations were about 0.001 Pa.s, identical to that of water. When the concentration of NaCl increased to the level of physiological saline, in which the concentration of NaCl is about 0.91 wt %, Laponite particles suspended in the aqueous solution began to aggregate and the viscosity of the system increased to about 0.003 Pa.s. Even in this high ionic strength, aggregated mass of Laponite in pure saline solution was still faint under CLSM, as shown in Figure 4g, indicating that the continuous network structure shown as red-light area in Figure 4h cannot be obtained in high ionic strength solution without HPMC. For the silanized formulations (pure Si-HPMC and Si-HPMC/XLS composite hydrogel), no Brownian motion could be observed as whole systems were crosslinked 2 h after preparation. It is shown in Figure 4h that fluorescent microspheres are well dispersed in the HPMC/XLS composite hydrogel, both in the red light area and dark area. The Brownian motion of fluorescent microsphere could be observed in the dark area from the CLSM movie. The local viscosity of the dark area in HPMC/XLS formulation was calculated at about 1 Pa.s, which is similar to that of pure HPMC solution, as shown in Figure 5, suggesting that in dark areas, HPMC solution is not aggregated with Laponite. On the contrary, in the red area the particles are “frozen”, which means that the red-light area is compact, or at least that holes are on the order of the tracers’ size (i.e., 1 µm). When flow is induced through a small pressure applied on the HPMC/XLS composite formulation, the fluorescent microspheres moved in the dark area along the flow direction, whereas they remain essentially frozen when they seat in the red area. This phenomenon reveals that the red area in the composite hydrogel was a dense area, which might contribute the high modulus to the whole system. Meanwhile, the dark area was a loose physical network area that forms an interconnected continuous space. 

### 3.5. Cell Behavior

Following the Physico-chemical investigations, we evaluated the cytotoxicity of our constructs (i) in 2D culture with the XLS suspension placed on the top of the cell layer, (ii) in 2D culture with the hydrogels that are placed above the cell layer, and (iii) in 3D culture with the cells embedded within the hydrogels.

**Figure 6:**
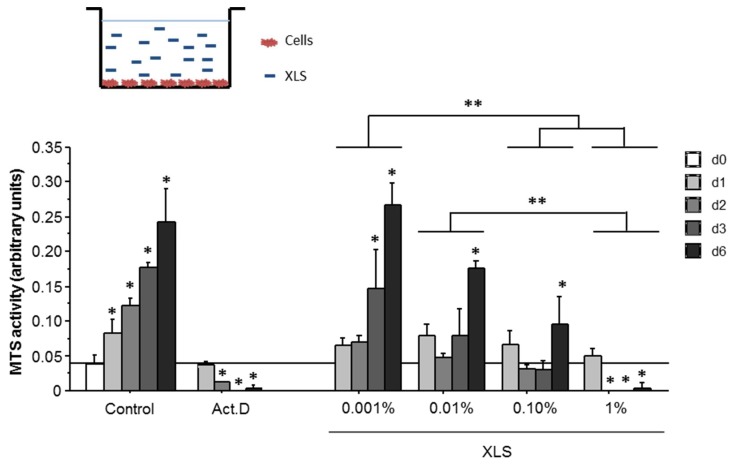
Methyl tetrazolium salt (MTS) activity of SW1353 cultured in two-dimensions (2D). (A) SW1353 viability was evaluated in 2D after adding 0–1% XLS, as indicated on top of the cell layer (10,000 cells/cm^2^). As described in materials and methods, a MTS assay was performed at day 0, 1, 2, 4, and 6. Negative control (Act.D) was obtained by growing SW1353 in the presence of actinomycin D (5 μg/mL). Statistics: * *p* < 0.01 vs. d0, ** *p* < 0.01 between the indicated groups.

As shown in Figure 6, an increase of the MTS activity SW1353 cells is observed from day 0 to day 6. When SW1353 cells are cultured in direct contact with XLS Laponites, their MTS activity was decreased at 0.01% XLS and above. Moreover, this decrease was correlated to the increase of the XLS concentration only from day 2 and onwards. Moreover, an increase in MTS activity is mainly observed at 0.001% of XLS Laponites from day 3 to 6. At higher concentrations (e.g., 0.01% and 0.1%), the increase can only be observed at day 6. At the higher concentration (1%), a decrease of the MTS activity was observed that is no longer different from the residual MTS activity that was measured when the cells are treated with actinomycin D (Act.D).

**Figure 7:**
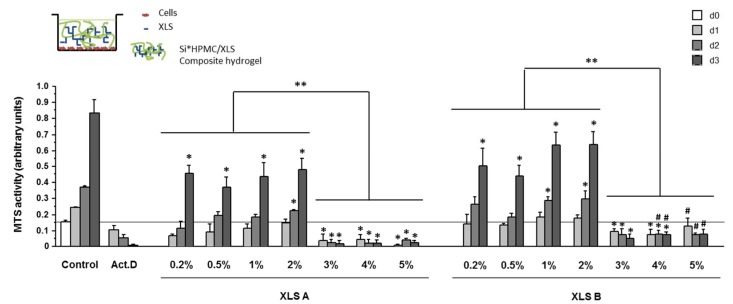
MTS activity of SW1353 cultured in 2D. hASC viability was evaluated in 2D after molding Si-HPMC hydrogels with 0 to 5% XLS (protocol A (XLS A) and B (XLS B)), as indicated on top of the cell layer (10,000 cells/cm2). As described in materials and methods, a MTS assay was performed at day 0, 1, 2, 3. Negative control (Act.D) was obtained by growing hASC in the presence of actinomycin D (5 μg/mL). * *p* < 0.01 vs. d0; ** *p* < 0.01 between the indicated groups; # *p* < 0.01 vs. XLS A at the same % and same day.

The MTS activity was also monitored in 2D with the cell layer in contact with the composite hydrogels (Si-HPMC/XLS). The results, which are plotted in Figure 7, showed a drastic decrease of MTS activity for Laponite concentration above 2%. 

These results showed an influence of the protocol used for the formulation that contain 1% XLS Laponites and above. A global significant decrease of the MTS activity was observed at all time points with protocol A as compared to B. 

From day 1 to 3, for all of the formulations with 0.2% to 2% of Laponites, MTS activity increased. At day 1, MTS activity was significantly lower at 0.2 and 0.5% XLS prepared with protocol A as compared to B. Then, the MTS activity significantly increased with protocol A at 1 and 2% of XLS, and then significantly decreased at 3 to 5% XLS. When prepared with B, MTS activity was not significantly different between 0.2 to 2% XLS, but decreased significantly for concentrations of XLS above 2%. 

At 3% of Laponites and above, MTS activity was significantly decreased when compared to control cells, regardless of the time of culture and was comparable to the residual MTS activity measured when the cells are treated with Act.D. 

**Figure 8:**
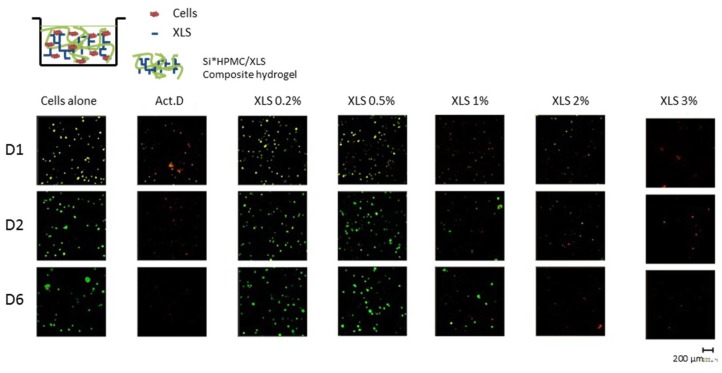
Three-dimensional (3D) viability of SW1353 cultured into Si-HPMC hydrogel containing increasing concentrations of XLS using the protocol B. SW1353 viability was evaluated in 3D after molding Si-HPMC hydrogels containing increasing concentrations of XLS (0–3%) and mixed with 1 × 10^6^ SW1353 at day 1, 2, and 6 by Live/Dead Cell Viability assay. Living cells were stained by Calcein AM in green and dead cells were stained in red by ethidium homodimer-1. Negative control was obtained by adding actinomycin D (Act.D; 5 μg/mL) in the culture medium. The pictures are representatives of all the 3D images of all the conditions, times, and levels of the hydrogels. Scale bar: 200 μm.

Following the 2D cytotoxicity assays evaluation, 3D cell proliferation was investigated (Figure 8). The confocal micrographs indicate a good viability of SW1353 cells cultured within Si-HPMC/XLS composite hydrogels up to 0.5 wt %. When cells were cultured in the presence of actinomycin-D or with high % of Si-HPMC/XLS composite hydrogels up (1% and above), dead cells are observed. These results evidence that at low concentrations of XLS (below 1%), the nanocomposite hydrogels have no negative effect on cells proliferation.

To understand the toxic effect of Laponites nanoparticles on the SW1553 2D cell in 2D culture, Laponites (at 1 wt %) were removed after 1 or 2 days, and cells were analyzed under Transmission Electron Microscopy. 

**Figure 9:**
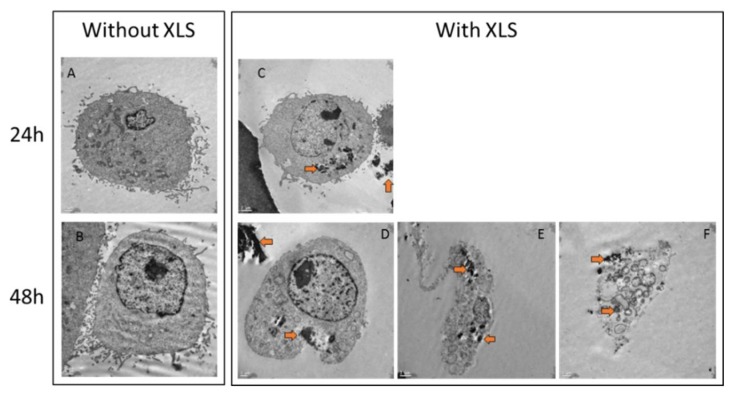
TEM micrographs of SW1353 cultured during one or two days into Dulbecco’s modified Eagle’s medium (DMEM) media containing XLS (**C**–**F**) or not (**A**,**B**). SW1353 fate was evaluated at 24 h (**A**,**C**) and 48 h (**B**,**D**–**F**). The samples were fixed, embedded, cut, and contrasted, as described in materials and methods. Aggregates of Laponites, appearing in black on TEM pictures (orange arrow), are internalized by cells and vesicles can be observed at 48 h. Scale bars correspond to 1 μm (**A**,**B**,**D**,**F**) and 2 μm (**C**,**E**).

Without Laponites, trypsinized cells showed round shape with distinct organelles and nucleus Figure 9A,B. After one day in contact with XLS Laponites, internalized aggregates of Laponites can be observed in the cytoplasm of cells (Figure 9C, orange arrows). After two days in contact with Laponites, cells started dying and living cells exhibited more internalized aggregates of Laponites in the cytoplasm and more vesicles (Figure 9D). Many cells are undergoing morphological changes with disorganized cytoplasm, loss of plasma membrane integrity, overall cell and nucleus shrinkage, numerous vacuoles, and vesicles containing Laponites aggregates (Figure 9E,F). These images are reminiscent of cells undergoing necrosis or autophagy. But, complementary experiments would be needed to discriminate their cell death pathway.

## 4. Discussion

### 4.1. Structure of the Hydrogel

The first part and aim of this study was to follow the influence of Laponite concentration on the structure of the formulations using two different preparation protocols. Rheological measurements were used, and it was observed that in the presence of HPMC or Si-HPMC a gel was formed. With HPMC a quite rapid increase of the modulus was seen before the first data point measurement and the evolution of the modulus was quite weak at longer times. On the contrary, the storage moduli of Si-HPMC and Si-HPMC/XLS formulations increased relatively gradually, but reached a higher value as compared to those of the HPMC/XLS formulations. These phenomena suggested that the formed network structures in HPMC formulation and Si-HPMC formulations might be different.

The modulus of HPMC/XLS system with 2 wt % XLS prepared using B without any covalent network structure could also reach a high value (about 780 Pa), which further indicated that some special structure might formed in hydrogel system that was prepared using B.

The mechanical spectra suggest that there is only a weak physical network structure in HPMC/XLS composite hydrogel system, while chemical network structures were formed in the Si-HPMC pure or composite hydrogels prepared using B.

As HPMC is an inert cellulose derivative without any reactive group on the polymer backbone, the pure HPMC solution showed a typical viscous flow behavior, as expected. When HPMC was silanized, silanol groups that were grafted onto the polymer chain could react with each other on decreasing pH, causing condensation reaction and forming chemical network structure [9]. When XLS was associated with HPMC solution using B, a physical network structure was shown to be formed as HPMC/XLS system behaved like a weak gel with narrow linear viscoelastic region, as shown in Figure 3. 

When Laponite particles are mixed with Si-HPMC, we can observe on Figure 1b that the addition of Laponite induces a sharp increase of the modulus of the gel above 1% (factor 2 for 1% and almost factor 10 for 2%). This increase is obviously not due to the simple addition of the moduli of both structures, but to a synergistic effect of Laponite and Si-HPMC chains. Indeed, XLS disks bear silanol groups on their edges [27,28], and these groups can condensate with silanol groups on Si-HPMC. Therefore, XLS dispersed in composite hydrogels can act as an additional crosslinking agent to increase the density of crosslinks and to improve the strength of the hydrogel, which could explain a large part of the increase of the composite hydrogels’ moduli with low content of XLS, as shown in Figure 1b. However, when the content of XLS was above 1 wt %, the modulus of composite hydrogel prepared using B increased sharply, being much higher than the sum value of pure Si-HPMC with chemical network structure and HPMC/XLS with pure physical network structure. We therefore proposed that a special bi-network structure that combines chemical and physical network structure together was formed in Si-HPMC/XLS composite hydrogel with 2 wt % XLS. In pure Si-HPMC hydrogel, only silanol groups on polymer long chains condensate with each other to form pure chemical network structure. When Laponite is introduced into the system, the individual XLS particles can attract each other and form compact aggregates due to the high ionic strength in the system, but can also attract polymer chains through adsorption mechanisms. If the concentration of XLS was as low as only 0.5 wt %, the aggregated particles do not reach the percolation threshold. When the concentration of XLS increases, Laponite aggregates, which contain polymer chains, can percolate, leading to a continuous network. This could explain why the modulus of HPMC/XLS(0.5 wt %) was too low to be observed, and why that of HPMC/XLS(2 wt %) could reach up to a relatively high value, as shown in Figure 1b. In this situation, a special double-network structure, somewhat similar to an interpenetrated polymer network (IPN), is formed where a chemical Si-HPMC network is reinforced by a physical XLS network, which can be chemically bound to polymer chains through the Si-O function of the Laponite particles. The synergistic effect between these two kinds of networks leads to the great improvement of modulus of composite hydrogel. Figure 3b shows the amplitude sweep curve of Si-HPMC/XLS composite hydrogel with 2 wt % XLS prepared using B. Altough this hydrogel resist to high deformations, around 1% strain a partial breakdown of physical bounds [29,30,31] can explain the slight drop in modulus. 

Figure 4e shows the image of Si-HPMC/XLS composite hydrogel with 1wt % of XLS prepared using protocol A. The image of HPMC/XLS system is similar to that of silanized systems (data not shown). It is obvious that different protocols led to different morphologies in the composite hydrogels. Protocol A led to a separated sea-island structure, while B resulted in a continuous irregular network structure. This can give a reasonable explanation for the results that are shown in Figure 1b, where the moduli of Si-HPMC/XLS composite hydrogels prepared using B are much higher than those of hydrogels that were prepared using protocol A. This continuous irregular network structure is required in order to obtain the high modulus composite hydrogels. 

The reason why the different protocols lead to the different structures in composite hydrogels can be explained, as follows. In protocol A, Laponite particles firstly meet high ionic strength buffer solution, which induces the immediate aggregation of particles and the formation of compact clusters. When polymer is later introduced, it is hard for the long polymer chains to penetrate into the clusters. Therefore, the polymer only adheres outside. The picture is simply that of essentially Laponite clusters that are bridged by Si-HPMC network. However, in B, Laponite particles meet polymer and electrolyte simultaneously. Then, the long polymer chains can connect with Laponite particles and react before they aggregate. When long polymer chains are added, the aggregation of Laponite in the hydrogel system that is prepared using B is totally different from that prepared using protocol A. 

**Figure 10:**
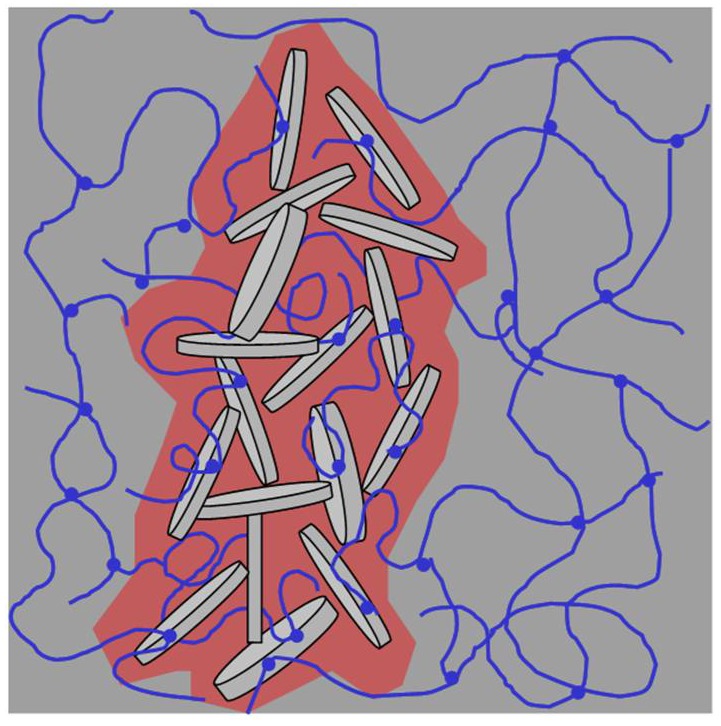
Illustration of possible double-network structure in silanized hydroxypropylmethylcellulose (Si-HPMC)/Laponite composite hydrogel. The curve line represents Si-HPMC polymer chain, the little plates represent Laponite and the round dots represent crosslink point where silanol condensation occurred. The silanols come from both Si-HPMC and Laponite. As Laponite has been dyed by Auramine O, the compact mass composed of aggregated Laponite and entangled polymer chain is in red under CLSM. It is likely that the XLS aggregates are more random than purely edge-faces contacts.

Figure 10 is a schematic illustration of the special bi-network structure in the Si-HPMC/XLS composite hydrogel that was prepared using B. 

There are two parts in this system: a dense Laponite rich part and Laponite free or loose part. As silanols on Laponite can react with silanol on cellulose, the long polymer chains can, not only adsorb onto Laponite particles, but also covalently bridge particles. In solution with high ionic strength, Laponite tend to aggregate. Then, the Laponite aggregates and linked polymer chains built a special compact network in the composite system. Even if there are many Laponite particles that are connected with long polymer chains, it is likely that the shape of the dense structure can be affected by the flow of polymer solution during the blending process. This could be the reason why the aggregated mass is irregular under CLSM. Meanwhile, the area outside the compact part is only polymer network and it is relatively loose. The compact network and loose native chemical network form co-continuous structures and the modulus of the sample is dominated by the modulus of the Laponite dense structure, leading to the high modulus of the composite hydrogel. The loose chemical network in the dark area is not changed with the addition of XLS, being similar with that of pure Si-HPMC hydrogel, which provides enough continuous space for the nutrient transportation and cell cultivation. Generally, scaffolds for tissue engineering should be highly porous, have adequate mechanical stability and strength, and contain interconnected pores to permit cell penetration and in-growth into the implanted structures [32,33]. These Si-HPMC/XLS composite hydrogels with high modulus and continuous room might be adapted biomaterials for specific tissue engineering strategies.

### 4.2. Cytotoxicity

Previous studies have demonstrated that internalization of Laponite nanoparticles induces cell death [34,35]. Other groups developing clay-based hydrogels also showed that cells remain viable when cultured with the biomaterials either in 2D or in 3D [36,37,38].

Here, we present evidence that free XLS Laponites (Figure 6) at concentration above 0.001% decrease metabolic activity of SW1353 cells, and after two days, we observed important cell death and internalization of Laponites aggregates in surviving cells (TEM analysis Figure 9). These observations suggest a toxic effect of XLS Laponites internalization in the SW1353 cells. We have also observed a drastic toxicity of the XLS from 3%, and onwards in Si-HPMC composite hydrogel whatever the protocol used (A or B) and the time in culture. 

In 2D with hydrogel above cell layer (Figure 7), at XLS concentration below 3% and when using protocol A, we observed a biphasic behavior of the cell. We have observed an initial decrease in the metabolic activity at day 1 and 2 for the 0.2 and 0.5% of XLS concentrations that is followed by a resumption of metabolic activity. This can be explained by slow kinetics of gelation (data not shown) that lead to an increase in the time of contact between Laponites and cells, and consequently to an increase in Laponites internalization at the early time points. Once the composite hydrogels are set, the structure is steady/stable and cells cannot internalize Laponites anymore. After three days, the remaining living cells resumed their growth, as illustrated by the increase in metabolic activity. 

Using protocol B, in 3D (Figure 8), we observed a decrease of living cells from 1% of XLS. Once cultured in 3D, the encapsulated cells are surrounded and in close contact with the Laponites, inducing an increase in their internalization, and consequently, a decrease of the surviving cells. Nevertheless, the few remaining living cells resume their growth at the later time points (Figure 8, 1% condition), leading to an increase in living cells at day 6.

Overall, our data demonstrated that a fine tuning of the kinetics of hydrogel/XLS composite gelation will be needed to minimize toxic effects XLS Laponites and cell internalization.

This study validates the IPN hybrid high modulus hydrogel for tissue engineering and animal model will then be used for the biocompatibility investigations of these new IPN hybrids.

## 5. Conclusions

A novel silanized hydroxypropylmethylcellulose/laponite composite hydrogel with great improvements in modulus has been prepared. Rheological experiments disclosed that there were both chemical and physical network structures in the composite hydrogel. Morphologies of Laponite dispersion were observed by CLSM directly and showed that different composite structures can be formed following different protocols. The Brownian motions of fluorescent microspheres in the systems revealed that there are two areas in the Si-HPMC/XLS composite hydrogel: compact areas, composed of aggregated Laponite and entangled polymer chains, both connected through covalent bonds and loose areas with a diluted crosslinked polymer network. This special double-network structure made this composite hydrogel system have great application potential in tissue engineering as biomaterials: The compact continuous network leads to the high modulus of the composite hydrogel, and the loose area provides continuous room for the nutrient transportation and cell cultivation. The biological investigations have clearly demonstrated toxicity of free XLS laponites. However, once the composite hydrogel is set, low toxicity is observed and the cells could resume their proliferation. The tuning of the kinetics of the setting process seems to be the critical parameter for the further use of these composite hydrogels for tissue engineering applications well adapted to be tested in long term for biocompatibility and biofunctionalities in different animal models for different applications.

## Supplementary Materials

The following are available online at http://www.mdpi.com/2073-4360/10/6/634/s1, Video S1: title. Video S1 Saline: CLSM movie of Fluorescent microspheres do Brownian motion in physiological NaCl solution (0.9 wt %). XLS appears red and fluorescent microspheres appear green under CLSM (Figure 4f & Viscosity in Figure 5). Video S2 XLS Water: Fluorescent microspheres in XLS suspension without ionic strength. XLS appears red and fluorescent microspheres appear green under CLSM (Viscosity in Figure 5). Video S3: XLS saline: Fluorescent microspheres in XLS suspension with high ionic strength. NaCl (0.1 M). XLS appears red and fluorescent microspheres appear green under CLSM (Figure 4g & viscosity in Figure 5). Video S4 HPMC: Fluorescent microspheres in pure HPMC solution (2 wt %). XLS appears red and fluorescent microspheres appear green under CLSM. (Viscosity in Figure 5). Video S5 HPMC XLS s: Fluorescent microspheres in HPMC/XLS composite system. HPMC (2 wt %), XLS (1 wt %). (Figure 4h & viscosity in Figure 5). Video S6 HPMC XLS f: Fluorescent microspheres in HPMC/XLS composite system under external pressure. HPMC (2 wt %), XLS (1 wt %). There is a little flow in it. XLS appears red and fluorescent microspheres appear green under CLSM. Video S7 Si-HPMC: Fluorescent microspheres in Si-HPMC pure hydrogel (2 wt %). FM was frozen. XLS appears red and fluorescent microspheres appear green under CLSM. Video S8 Si-HPMC XLS: Fluorescent microspheres in Si-HPMC/XLS composite system. Si-HPMC (2 wt %), XLS(1 wt %). Fluorescent microspheres was frozen. *All the above samples were filmed after being prepared for 2 h.
